# Simulation-based education improves student self-efficacy in physiotherapy assessment and management of paediatric patients

**DOI:** 10.1186/s12909-019-1894-2

**Published:** 2019-12-16

**Authors:** Judith Hough, Daniel Levan, Michael Steele, Kristine Kelly, Megan Dalton

**Affiliations:** 1 School of Allied Health, Australian Catholic University, Banyo, Queensland Australia; 2 Mater Research – The University of Queensland, South Brisbane, Queensland Australia; 3 Office of the Executive Director of Allied Health, South Brisbane, Queensland Australia

**Keywords:** Simulation, Paediatrics, Physiotherapy

## Abstract

**Background:**

The Australian Physiotherapy Council mandates that physiotherapy clinical education be sufficient to produce graduates who are competent to practice across the lifespan. Due to a lack of opportunities for paediatric clinical placements, there is a risk of graduates not having the opportunity to develop competency in paediatric physiotherapy. To address this risk, simulation-based education (SBE) has been proposed as an educational strategy to address the placement shortfall. Despite encouraging evidence for its use in physiotherapy education, there is limited evidence supporting its use specifically in paediatric populations. The aims of this research were to investigate the effect of SBE on student self-efficacy in the physiotherapy assessment and management of paediatric clients, and to determine student satisfaction with SBE as a learning strategy.

**Methods:**

Three interactive SBE sessions were run during the undergraduate paediatric physiotherapy unit at the campus of one Australian university. Self-efficacy was surveyed before and after each session, to determine confidence in clinical skills, clinical decision-making, treatment preparation and planning, communication skills; evaluating and modifying interventions, and interprofessional practice. Student satisfaction with SBE as a learning strategy was surveyed after the final SBE session.

**Results:**

For the 164 participants included in this study, self-efficacy survey response rate varied from 77 to 96% for each session. Significant increases in mean student self-efficacy were recorded for all questions (*p* <  0.001). A total of 139 (85%) responded to the learning reactionnaire with 78.6% indicating they were very satisfied with SBE as a learning strategy. Written comments from 41 participants identified ‘experience’ as the primary theme.

**Conclusion:**

SBE had a significant positive effect on student self-efficacy in the physiotherapy assessment and management of paediatric patients. Students also perceived SBE to be a valuable learning experience. Future research is needed to investigate whether the improvement in self-efficacy achieved through SBE translates into improved student performance during workplace-based clinical placements.

## Background

In physiotherapy undergraduate courses, clinical education constitutes approximately a third of the program, with students spending between 900 and 1000 h in supervised clinical time with patients. This professional practice component is required for program accreditation by the Australian Physiotherapy Council (APC) [1]

Clinical education relies on the availability of a diverse range of patients, however the scenarios students are exposed to, vary depending on the patients presenting during the placement [2]. Individual learning experiences vary in this model, creating learning environments which may be opportunistic, unstructured and disparate [3] Furthermore, students may get inadequate exposure to higher risk patients, resulting in missed learning opportunities [4].

In addition to these challenges, paediatric placements are limited due to a lack of suitably qualified paediatric physiotherapists to act as educators [5]. Students may therefore not have the opportunity to develop competency in paediatric physiotherapy practice prior to registration as a physiotherapist, despite the APC’s requirement for development of competency across the lifespan [1].

To address the limitations of clinical education in general, and more specifically in paediatric physiotherapy education, simulation-based education (SBE) has been proposed as an additional educational strategy. SBE attempts to replicate real-life experiences through simulated scenarios, environments or patients, creating a safe environment where clinical confidence and competence can be developed [6, 7]. With SBE, learning experiences can be tailored to specific learning objectives and can be set up on demand, eliminating the dependence on patient availability [2]. If implemented successfully, SBE can guarantee provision of consistent and diverse learning experiences and include exposure to scenarios that are clinically uncommon, promoting a more equitable learning experience for all students.

SBE has been shown to be an effective method for learning a range of physiotherapy skills, including hands-on [2, 8–19] and interdisciplinary skills (including teamwork and communication) [20–27]. Up to 25% of clinical placement experiences may be replaced by SBE without compromising student learning [28, 29]. Changes to student attitudes have also been reported following SBE [30–33], including improved motivation to learn [30] and improved awareness of physiotherapy core values [32, 33]. Students have generally viewed SBE as a positive learning experience [2, 14, 20, 25, 34–40] and while this does not equate to an actual learning effect, it may influence motivation to learn [41].

Improved levels of student self-efficacy have also been demonstrated with SBE [28, 29, 32, 36–40, 42, 43], where self-efficacy is defined as an individual’s personal judgment in their own ability to successfully accomplish a task [44]. Self-efficacy is a key attribute in professional practice as there is a demonstrated relationship between self-efficacy and work-related performance, such as performance in clinical environments [45].

Despite this encouraging evidence for the use of SBE in physiotherapy education, there is insufficient evidence supporting the use of SBE specifically in paediatric populations. Considering the limited clinical learning opportunities available in paediatric physiotherapy, it is important for alternative methods of education to be considered and evaluated. Therefore, the primary aim of this study was to investigate the effect of SBE on student self-efficacy in the physiotherapy assessment and management of paediatric clients. A secondary aim of this study was to determine student satisfaction with SBE as a learning strategy.

## Methods

This study was a prospective, observational study using self-efficacy questionnaires and a learning reactionnaire to survey student satisfaction with SBE as a learning strategy.

### Participants

Students studying physiotherapy at an Australian university campus were eligible for admission to the study if they were enrolled in the Paediatric Physiotherapy Practice academic unit of study which was delivered in the 3rd year of their physiotherapy program and was the final unit prior to their clinical placement year. The study was undertaken in 2014 (the first year of simulation delivery) and in the exact same format in 2018 (the most recent year to determine if there was evidence for ongoing delivery of SBE). Students who had previously attended any classes in the Paediatric Physiotherapy Practice unit (or equivalent) were excluded from the study.

Written consent was obtained prior to commencement of the first scenario. Human Research Ethics clearance was obtained through the institute HREC committee (2018-56E).

### Outcome measures

The primary outcome measured in this research project was student self-efficacy in the physiotherapy assessment and management of paediatric patients. Student self-efficacy was measured using a self-efficacy questionnaire developed by Health Workforce Australia and used throughout Queensland by the Simulated Learning in Paediatric Allied Health (SLIPAH) team. This questionnaire was developed in 2010 by SLIPAH in collaboration with Clinical Skills Development Service (CSDS), a Queensland Government training provider (https://csds.qld.edu.au/). The questionnaire was designed to evaluate the efficacy of SBE against the second level (learning) of Kirkpatrick-Phillips’ model of training evaluation, an established framework for the evaluation of training programs [46]. The second level of *learning* refers to changes in knowledge, skills or attitudes and is often described as a transfer of knowledge. It does not include behavioural changes or practical application resulting from these changes in knowledge, skills or attitudes [46].

Student satisfaction with SBE as a learning strategy was the secondary outcome of this project and was measured using a learning reactionnaire. This learning reactionnaire was adapted by the SLIPAH team from designs initially published by Leslie Rae [47], and was intended to establish student levels of engagement with SBE. This evaluates the first level (reaction) of Kirkpatrick-Phillip’s model of training evaluation, which refers to how trainees reacted to the training [46]. Both questionnaires used a Likert 5-point rating scale, ranging from 0 (not at all confident/learning nothing) to 4 (totally confident/learned a lot). The learning reactionnaire also gave participants the opportunity to add comments to provide additional feedback on their experience.

### Study procedure

**Fig. 1:**
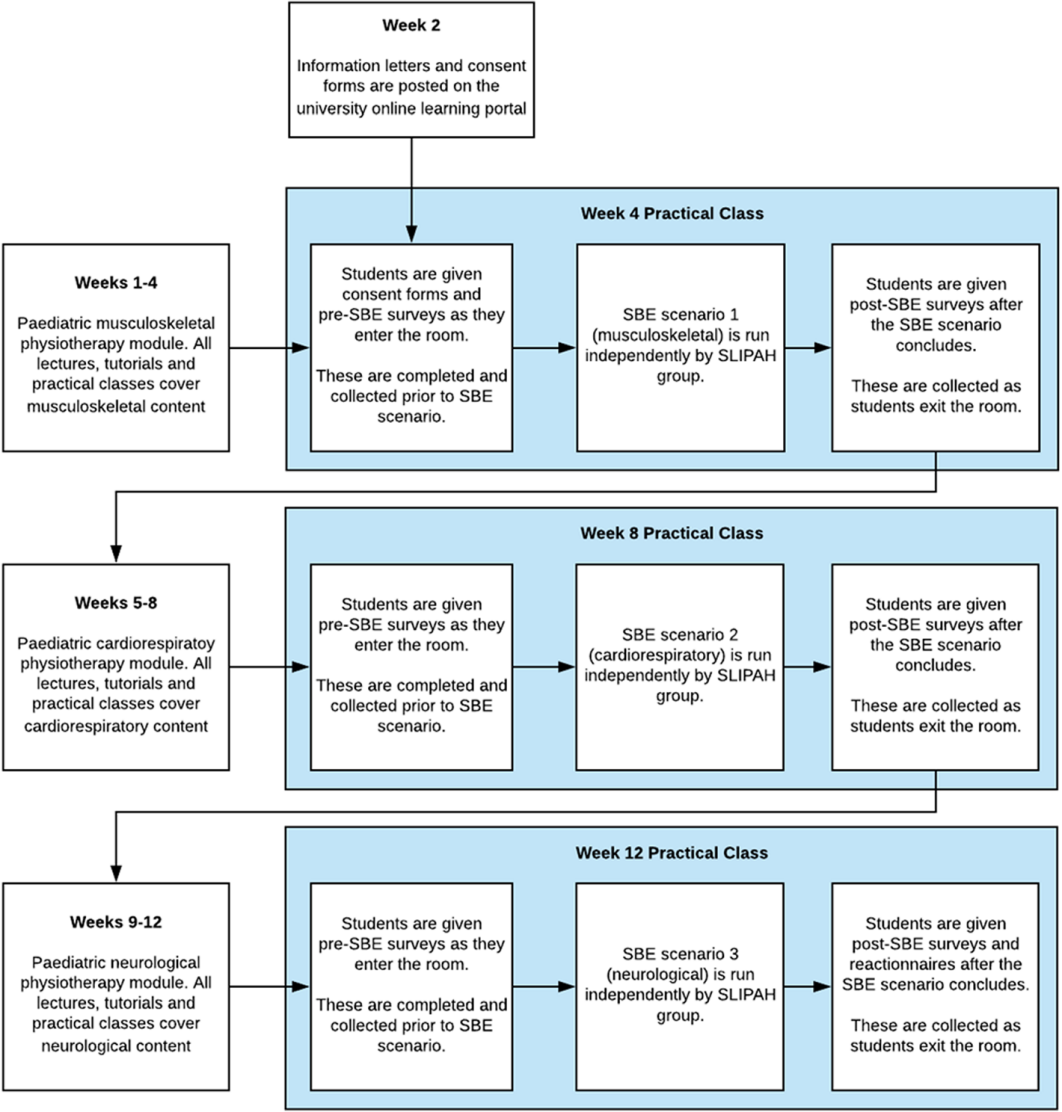
Timeline of study procedure

Each SBE session was designed and conducted by the SLIPAH group in consultation with university teaching staff and form part of the regular academic unit curriculum during the Paediatric Physiotherapy Practice practical classes in weeks 4, 8 and 12 of a 12-week semester (Fig. 1). The scenario for each SBE session specifically targeted one of the primary clinical domains of paediatric physiotherapy and was delivered with the corresponding musculoskeletal, cardiorespiratory and neurodevelopmental modules of the unit. In the week prior to the SBE session, the students were requested to independently undertake an eLearning package to prepare them for each session (https://www.sdc.qld.edu.au/). The three eLearning packages suggested were on General Allied Health Paediatric Principles, Cardiorespiratory acute paediatric physiotherapy, and spina bifida and spinal disabilities.

The interactive SBE sessions used high fidelity paediatric human patient simulators which are life-like, anatomically correct, computer driven mannequins with physiologic responses that mimic real patients. For the musculoskeletal and cardiorespiratory scenarios, the SimJunior® (Laerdal Medical, Victoria, Australia) mannequin was used and for the neurodevelopmental scenario, it was Sophie Newborn® (Laerdal Medical). Each SBE learning scenario was conducted over a two-hour period with 25 students per group. There was a 1:8 ratio of students to educators in each group.

Self-efficacy questionnaires were completed by participants in the first 5 min of each session and were collected prior to commencement of the scenario. A second, identical questionnaire was given to each participant at the completion of the SBE scenario. At the completion of the third and final SBE session the learning reactionnaire was also given to participants and collected as students left the session. All questionnaires were given in paper format.

### Statistical analysis

To analyse the pre-post differences in self-efficacy for each of the three scenarios, a Wilcoxon Signed Ranks Test and a descriptive statistics report was undertaken using IBM SPSS Version 25 (IBM Corp, Armonk, NY, USA). A Kruskal Wallis test was used to determine if there were any differences between the 3 scenarios at baseline. A *p*-value of 0.05 was considered significant.

To report student satisfaction with SBE, descriptive statistics were used to report on quantitative data and a thematic analysis with Leximancer software Leximancer (Leximancer Pty Ltd., Brisbane, Australia) was used to report qualitative data.

## Results

From the cohort of ninety-two (92) students enrolled in the Paediatric Physiotherapy Practice unit in 2018, 1 student was excluded due to previous enrolment and 1 student declined to participate. Seventy-four (74) students were enrolled in the Paediatric Physiotherapy Practice unit in 2014. Questionnaire response rates for each of the three SBE scenarios and learning reactionnaire are in Table 1.

**Table 1:** Response rates to the learning reactionnaire and questionnaire for each of the three simulation-based education scenarios

	2014	2018
	*N* (%)	*N* (%)
Total enrolled	74	92
Scenario 1 - Musculoskeletal	70 (95)	86 (96)
Scenario 2 - Cardiorespiratory	67 (91)	82 (91)
Scenario 3 - Neurological	57 (77)	77 (86)
Learning Reactionnaire	62 (84)	77 (86)

### Self-efficacy

Significant increases in mean student self-efficacy were recorded for all questions in each scenario (*p* <  0.001) with improvements in mean self-efficacy scores ranging from 0.73–0.97 for all questions across all scenarios (Tables 2, 3, 4). Question 2 (clinical decision-making) had the largest mean improvement to self-efficacy in 2 out of the 3 scenarios (musculoskeletal and cardiorespiratory) as well as the largest mean improvement overall (0.93), while question 4 (maintaining communication with nurse, carer and child) had the lowest mean improvement in 2 out of the 3 scenarios (musculoskeletal and cardiorespiratory) as well as the lowest mean improvement overall (0.77). Questions 4 and 7 had the highest pre and post SBE scores in all 3 scenarios, and respectively had the highest pre (1.99, 1.91) and post (2.76, 2.72) scores overall. Question 2 had the lowest pre and post SBE scores in every scenario and overall (pre = 1.48, post = 2.41).

**Table 2:** Change in self-efficacy after SBE scenario 1 (musculoskeletal) for combined 2014 and 2018 cohort, where 0 = “not at all”, 1 = “a little”, 2 = “moderately”, 3 = “a lot” and 4 = “totally” confident

Question	Mean Pre Score	Mean Post Score	Mean Difference	*P*-Value	Negative Ranks	Positive Ranks	Ties	Total
Q1	1.54 ± 0.57	2.48 ± 0.62	0.94	< 0.001	0 (0%)	126 (80.8%)	30 (19.2%)	156
Q2	1.48 ± 0.61	2.45 ± 0.66	0.97	< 0.001	1 (0.6%)	122 (78.7%)	32 (20.6%)	155
Q3	1.71 ± 0.65	2.66 ± 0.62	0.95	< 0.001	1 (0.6%)	116 (74.8%)	38 (24.5%)	155
Q4	2.04 ± 0.72	2.77 ± 0.71	0.73	< 0.001	4 (2.6%)	99 (63.5%)	53 (34%)	156
Q5	1.67 ± 0.69	2.54 ± 0.62	0.86	< 0.001	3 (1.9%)	109 (69.9%)	44 (28.2%)	156
Q6	1.56 ± 0.70	2.48 ± 0.61	0.92	< 0.001	2 (1.3%)	118 (75.6%)	36 (23.1%)	156
Q7	2.01 ± 0.76	2.78 ± 0.66	0.76	< 0.001	4 (2.6%)	98 (62.8%)	54 (34.6%)	156

**Table 3:** Change in self-efficacy after SBE scenario 2 (cardiorespiratory) for combined 2014 and 2018 cohort, where 0 = “not at all”, 1 = “a little”, 2 = “moderately”, 3 = “a lot” and 4 = “totally” confident

Question	Mean Pre Score	Mean Post Score	Mean Difference	*P*-Value	Negative Ranks	Positive Ranks	Ties	Total
Q1	1.55 ± 0.61	2.43 ± 0.63	0.88	< 0.001	1 (0.7%)	110 (73.8%)	38 (25.5%)	149
Q2	1.49 ± 0.61	2.38 ± 0.65	0.89	< 0.001	2 (1.3%)	107 (71.8%)	40 (26.8%)	149
Q3	1.67 ± 0.64	2.52 ± 0.62	0.85	< 0.001	2 (1.4%)	107 (72.3%)	39 (26.4%)	148
Q4	1.98 ± 0.72	2.76 ± 0.67	0.78	< 0.001	3 (2%)	97 (65.1%)	49 (32.9%)	149
Q5	1.60 ± 0.63	2.45 ± 0.67	0.85	< 0.001	0 (0%)	107 (71.8%)	42 (28.2%)	149
Q6	1.58 ± 0.63	2.40 ± 0.69	0.81	< 0.001	3 (2%)	102 (68.5%)	44 (29.5%)	149
Q7	1.85 ± 0.70	2.71 ± 0.64	0.86	< 0.001	0 (0%)	104 (69.8%)	45 (30.2%)	149

**Table 4:** Change in self-efficacy after SBE scenario 3 (neurological) for combined 2014 and 2018 cohort, where 0 = “not at all”, 1 = “a little”, 2 = “moderately”, 3 = “a lot” and 4 = “totally” confident

Question	Mean Pre Score	Mean Post Score	Mean Difference	*P*-Value	Negative Ranks	Positive Ranks	Ties	Total
Q1	1.55 ± 0.58	2.41 ± 0.66	0.86	< 0.001	2 (1.5%)	98 (73.1%)	34 (25.4%)	134
Q2	1.47 ± 0.61	2.40 ± 0.65	0.93	< 0.001	2 (1.5%)	101 (75.4%)	31 (23.1%)	134
Q3	1.67 ± 0.64	2.56 ± 0.63	0.89	< 0.001	1 (0.7%)	96 (71.6%)	37 (27.6%)	134
Q4	1.96 ± 0.67	2.74 ± 0.68	0.79	< 0.001	3 (2.2%)	86 (64.2%)	45 (33.6%)	134
Q5	1.67 ± 0.56	2.44 ± 0.61	0.77	< 0.001	1 (0.8%)	90 (68.7%)	40 (30.5%)	131
Q6	1.56 ± 0.62	2.51 ± 0.73	0.95	< 0.001	2 (1.5%)	100 (74.6%)	32 (23.9%)	134
Q7	1.86 ± 0.67	2.67 ± 0.68	0.81	< 0.001	1 (0.7%)	92 (68.7%)	41 (30.6%)	134

**Fig. 2:**
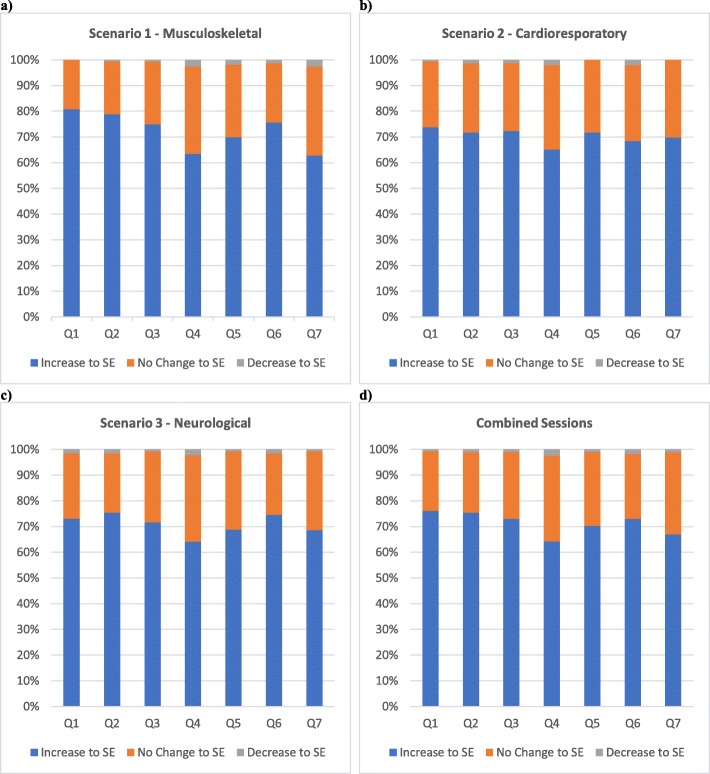
Percentage of students who had an increase, decrease or no change to self-efficacy (SE) for each session. **a** shows results for the first (musculoskeletal) scenario. **b** shows results for the second (cardiorespiratory) scenario. **c** shows results for the third (neurological) scenario. **d** shows results for all scenarios combined

In every question, participants who reported an increase in self-efficacy post SBE (positive ranks) outnumbered participants who reported a decrease (negative ranks) or no change (ties). For all questions across all scenarios: positive ranks ranged from 62.8–80.8% of respondents; negative ranks ranged from 0 to 2.6% of respondents; and ties ranged from 19.2–34.6% of respondents. (Figure 2) Question 1 (preparation for treating paediatric patients) and Question 2 (clinical decision-making) had the highest number of positive ranks recorded in 2 out of the 3 scenarios (musculoskeletal and cardiorespiratory), while Question 4 (maintaining communication) had the highest number of negative ranks and ties in 2 out of the 3 scenarios (cardiorespiratory and neurological). Question 4 also had the highest number of total negative ranks [9] and ties (147) recorded across all scenarios (Table 5).

**Table 5:** Change in self-efficacy for all sessions combined, where 0 = “not at all”, 1 = “a little”, 2 = “moderately”, 3 = “a lot” and 4 = “totally” confident

Question	Mean Pre Score	Mean Post Score	Post scores ≥ 3	Mean Difference	*P*-Value	Negative Ranks	Positive Ranks	Ties	Total
Q1	1.55 ± 0.59	2.45 ± 0.63	196 (44.6%)	0.90	< 0.001	3 (0.7%)	334 (76.1%)	102 (23.2%)	439
Q2	1.48 ± 0.61	2.41 ± 0.65	192 (43.8%)	0.93	< 0.001	5 (1.1%)	330 (75.3%)	103 (23.5%)	438
Q3	1.68 ± 0.64	2.58 ± 0.62	240 (54.9%)	0.90	< 0.001	4 (0.9%)	319 (73%)	114 (26.1%)	437
Q4	1.99 ± 0.71	2.76 ± 0.69	293 (66.7%)	0.77	< 0.001	10 (2.3%)	282 (64.2%)	147 (33.5%)	439
Q5	1.65 ± 0.64	2.48 ± 0.63	195 (44.7%)	0.83	< 0.001	4 (0.9%)	306 (70.2%)	126 (28.9%)	436
Q6	1.57 ± 0.65	2.46 ± 0.68	204 (46.5%)	0.89	< 0.001	7 (1.6%)	320 (72.9%)	112 (25.5%)	439
Q7	1.91 ± 0.72	2.72 ± 0.66	277 (63.1%)	0.81	< 0.001	5 (1.1%)	294 (67%)	140 (31.9%)	439

Analysis of the pre self-efficacy scores between the three scenarios showed no significant differences (*p* > 0.07).

### Student satisfaction with SBE as a learning strategy

There was a total of 139 responses to the learning reactionnaire, 62 (83.8%) in 2014 and 77 (85.6%) in 2018 (Table 6). Most student responses (78.6%) indicated that the SBE scenarios were an effective model for promoting learning in the field of paediatric physiotherapy. Mean responses ranged from 2.76 to 3.12 for all questions. The mean rating for Question 2 (promotion of self-directed learning) of 2.76 was substantially lower than the other questions, which ranged from 3.01–3.12, For the entire questionnaire, there was only 1 response with a rating of 0 (0.1%) and 14 recorded ratings of 1 (1.7%).

**Table 6:** Summary of learning reactionnaire responses for combined 2014 and 2018 cohort

Question	Score 0 (Not at all)	Score 1 (A little)	Score 2 (Moderately)	Score 3 (A lot)	Score 4 (Totally)	Range	Mean	Total
Q1	0 (0%)	1 (0.7%)	27 (19.6%)	80 (58%)	30 (21.7%)	3	3.01 ± 0.67	138
Q2	0 (0%)	7 (5.1%)	38 (27.5%)	74 (53.6%)	19 (13.8%)	3	2.76 ± 0.75	138
Q3	0 (0%)	1 (0.7%)	27 (19.6%)	64 (46.4%)	46 (33.3%)	3	3.12 ± 0.74	138
Q4	0 (0%)	2 (1.5%)	26 (19%)	75.5 (55.1%)^a^	34.5 (25.2%)^a^	3	3.03 ± 0.71	137
Q5	0 (0%)	2 (1.5%)	22 (16.1%)	72 (52.6%)	41 (29.9%)	3	3.11 ± 0.71	137
Q6	1 (0.7%)	1 (0.7%)	23 (16.7%)	74 (53.6%)	39 (28.3%)	4	3.08 ± 0.74	138
Total	1 (0.1%)	14 (1.7%)	163 (19.7%)	439.5 (53.1%)	209.5 (25.3%)	4	3.02 ± 0.73	827

^a^1 participant submitted a score of 3.5 for question 4

**Fig. 3:**
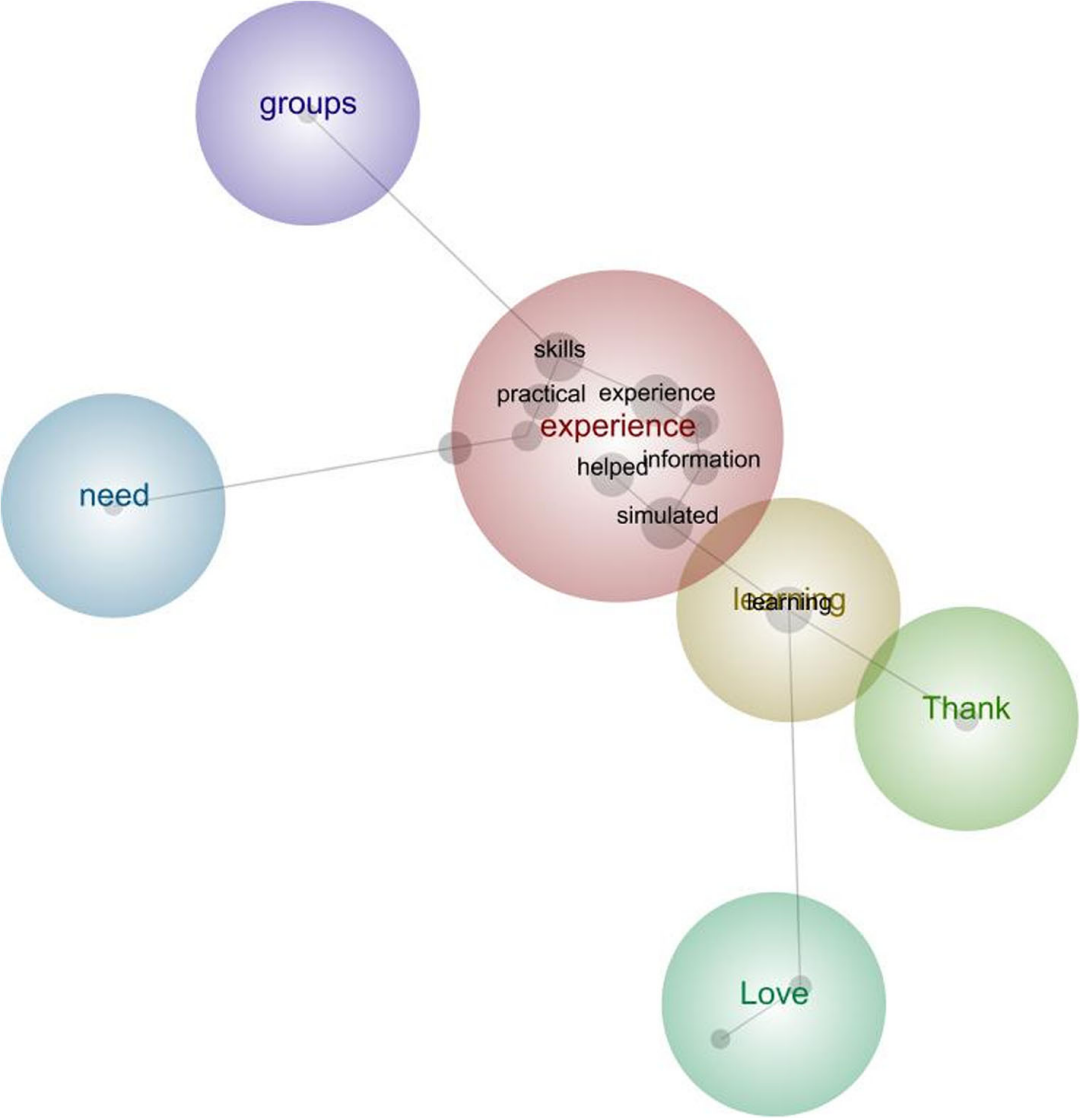
Leximancer concept map illustrating the main concepts identified by the students in the learning reactionnaire and how they interrelate with each other

Of the 62 learning reactionnaire responses submitted in 2014, there were 20 (32.3%) written comments, and for the 77 in 2018, there were 21 (27.3%) written comments. The primary theme identified was experience (reported in 13 (32%) comments) and was mentioned alongside the concepts of skills, practical, helped, information, and simulated (Fig. 3). Some examples of responses submitted included ‘Good *experience* + *practical skills;* Awesome learning *experience!;* They *helped* to put theory into practice and increase knowledge, understanding and *practical skills*; Very hands on and *practical;* The *simulated* classes were very *helpful* and helped with retention of *information;* Really good team, able to teach all the *information* effectively in a form that will be retained!; I like the handling *skills* we learnt and how to talk to other people in the team and the family.”

Other themes mentioned included learning [7], thank [5], need and groups [3], and love [2], with responses such as ‘Fantastic module! *Love* this style of *learning*; *Thank* you! It was a Great *learning* environment; Working in small *groups* with dolls was very beneficial; *Love* this way of teaching. It’s more real world.

## Discussion

Significant improvements in student self-efficacy in the physiotherapy assessment and management of paediatric clients was found with SBE, demonstrating that students felt improved confidence in clinical skills, clinical decision-making, treatment preparation and planning, communication skills, evaluating and modifying interventions and interprofessional practice. These improvements mirror previous research conducted in an adult physiotherapy context [36, 37, 42].

Students showed the largest improvements in Question 2, indicating that students perceived the greatest improvements to their clinical decision-making skills. Interestingly, this question had the lowest pre and post SBE scores in all scenarios, demonstrating that despite the improvement, students remained least confident in their clinical decision-making skills. Students have had limited opportunities to refine their clinical decision-making skills at this stage of their learning, which is a possible explanation for their lower initial levels of confidence. Given this lack of experience, it is reasonable that a small amount of experience (such as a single SBE session) would be sufficient to cause a significant increase in confidence.

Conversely, Questions 4 and 7 had the highest pre and post SBE scores, while having the smallest overall improvements in self-efficacy. Students were most confident in their communication and inter professional skills and perceived the least amount of improvements to these skills. Previous research has also shown students to have higher levels of confidence in their communication skills compared to their confidence in treatment and hazard awareness [43]. It is likely that students were more confident with their communication and interpersonal skills prior to SBE as they have had the more opportunities to develop these skills throughout the early years of their program of study. Furthermore, it is possible that additional learning experiences may be required to realise changes in these more well-developed skills. It is also possible that these smaller improvements to communication and interpersonal skills can be explained by a ceiling effect, given students’ higher initial levels of confidence.

A lack of confidence working with children has been reported to be a barrier to graduates seeking employment in paediatric physiotherapy, and evidence suggests confidence in graduate paediatric physiotherapists is directly related to competence in communication skills [48]. Therefore, confidence in communication is particularly important for graduates seeking employment as a paediatric physiotherapist. Although the questions relating to communication skills [4, 7] had the smallest improvements in self-efficacy, they were still statistically significant and the majority of students (64.9%) reported scores of 3 or higher after SBE, indicating that they had at least “a lot” of confidence in their communication and inter professional skills. It seems that SBE has provided a level of confidence in communication skills which may aid reduction of the barriers to working in a paediatric context.

Although self-efficacy improved from the start to the end of each SBE scenario, there was no improvement in pre self-efficacy scores over the course of the entire academic unit. At the start of each new SBE scenario, self-efficacy scores returned to baseline levels. As previously described by Wright et al. (43), this suggests that students’ self-efficacy is linked to area-specific knowledge and skills and does not necessarily transfer between areas. It is not a function of simulation, but the specific clinical knowledge and skills gained during the process.

### Student satisfaction with SBE

The secondary aim of this research project was to determine student satisfaction with SBE as a learning strategy. The response was positive, with most students considering that SBE met their style of learning, promoted self-directed learning, delivered evidence-based principles of paediatric physiotherapy, assisted in retention of paediatric physiotherapy, provided an ideal learning environment, and provided incentive for further skill development in paediatric physiotherapy. Only one student (0.7%) thought that SBE provided no incentive at all for further skill development in paediatric physiotherapy. These findings are consistent with two systematic reviews, which concluded that SBE is generally well received by students and an experience valuable to learning [2, 16]. The positive findings observed in the quantitative results were reflected by students’ comments. The thematic analysis identified. that students found SBE to be a beneficial and enjoyable learning experience.

The mean rating for Question 2 (promotion of self-directed learning) of 2.76 was substantially lower than the other questions, and again may reflect the structure of the SBE scenarios and/or insufficient emphasis on self-directed learning activities.

Overall, the student response to SBE was positive, and if students respond well to this method of learning, it could suggest they are more likely to be engaged with the learning experience and be more motivated to learn [41].

### Translation to performance

While the improvements to self-efficacy observed in this study are significant, these improvements may not translate to an improved level of workplace-based clinical performance. The self-efficacy questionnaires provide a measure of perceived confidence in students’ knowledge, skills and attitudes, demonstrating that they have achieved a degree of *learning*, according to the Kirkpatrick-Phillips’ model of training evaluation [46]. The questionnaires give no indication whether participants have applied what they’ve learned through changes in *behaviour*, the next tier in the Kirkpatrick-Phillips’ model [46]. There are well-established links between self-efficacy and work-related performance [45] so it is reasonable to suggest that students who demonstrated improvements in self-efficacy following SBE would have improved performance following SBE. Previously, SBE has led to significant improvements in student performance, [28, 43] improved patient care and better patient outcomes, [49] strengthening the suggestion that these improvements to self-efficacy may translate to improved performance with real patients. However, students who receive SBE also may be more likely to overestimate their ability [50] and are likely to be less realistic in their self-evaluation in a simulated environment [32]. Therefore translation to improved performance should not be assumed based on these self-reported measures alone.

To measure performance, student behaviour would need to be evaluated in a standardised or clinical environment. There are established instruments for measuring performance, such as the validated Assessment of Physiotherapy Performance (APP) [51] or the Physical Therapy Clinical Performance Instrument (PT CPI) [52]. The APP is currently used by Australian universities to evaluate the performance of students on clinical placement and has been used in other studies examining the efficacy of SBE [28, 29, 43, 50, 53]. In two previous studies, students who participated in SBE achieved superior grades, measured through their performance in the APP [28, 43]. It would be beneficial to conduct a follow-up study to determine if there are similar differences in student performance for this student group.

### Limitations

Although clear improvements to student self-efficacy following SBE were observed in this study, there is no comparison to standard educational methods. Without this comparison, it is not possible to determine whether the educational benefit gained from SBE differs from standard curriculum. This is especially important given the high cost of SBE, which is often a barrier to its implementation [54].

Students completed their self-efficacy questionnaires immediately after the SBE scenarios ended, meaning that the results only reflect the short-term effect of SBE. Although some students commented that they felt SBE improved their retention of paediatric physiotherapy, without further investigation it is not possible to determine if SBE had any long-lasting effects. There is also no measure on actual student performance.

By comparing APP results of paediatric placements, we could investigate if there is any difference between the performance of students who participated in SBE and those who received standard curriculum, as well as gain information on the long-term effects of SBE, as clinical placements occur months after the SBE ends.

There is no information on whether SBE changed students desire to work in paediatric physiotherapy in the future. Results from learning reactionnaire Question 6 (incentive to develop skills further in paediatric physiotherapy) offers some support for this, with 82% responses recorded as “a lot” or “totally”, demonstrating that students had incentive to further develop paediatric physiotherapy skills following SBE. However, this does not mean students would pursue paediatric physiotherapy opportunities. To get a better indication of this, it would be beneficial to ask students if SBE has increased the likelihood of them pursuing a career in paediatric physiotherapy or requesting a paediatric placement in future research in this area.

Only undergraduate university students were included in this study, so results should not be extrapolated and applied to other populations such as junior, employed physiotherapists. Furthermore, participants were all from one campus of one Australian university and may not accurately represent students of other campuses or Australian universities.

## Conclusion

The results of the self-efficacy questionnaire were clearly positive, with significant improvements to student self-efficacy post SBE recorded for every question, indicating that SBE has a positive effect on student self-efficacy in the physiotherapy assessment and management of paediatric clients. Students also reported that they found SBE to be a valuable learning experience.

## Data Availability

The datasets used and/or analysed during the current study are available from the corresponding author on reasonable request.

## Abbreviations

ACU: Australian Catholic University
APC: Australian Physiotherapy Council
APP: Assessment of Physiotherapy Performance
CSDS: Clinical Skills Development Service
HREC: Human Research Ethics Committee
PT CPI: Physical Therapy Clinical Performance Instrument
SBE: Simulation-based education
SE: Self-efficacy
SLIPAH: Simulated Learning in Paediatric Allied Health
